# 

**Published:** 1914-09

**Authors:** 


### CONTENTS.

ORIGINAL COMMUNICATIONS—

The Value of Epididymotomy, by E. P. Merritt, M. D., Urologist to the Atlanta Anti-Tuberculosis Association and Assistant in Urology at the Atlanta Medical College .......... 241

A Plea for the Conservation of the Inferior Turbinate, by Albert B. Mason, M. D., Waycross, Ga. .......... 245

The Diagnosis of Surgical Affections of the Kidney and Bladder, by Edgar G. Ballenger, M. D., and Omar F. Elder, M. D., Atlanta .......... 250

Meeting of the Fulton County Medical Society, by R. R. Daly, M. D. .......... 257

EDITORIAL .......... 266

SELECTIONS AND ABSTRACTS .......... 270

BOOK REVIEWS ..........

